# Decoding the Prognostic Significance and Therapeutic Implications of Inflammation-Based Scores in Hepatocellular Carcinoma: A Comprehensive Review

**DOI:** 10.3390/cancers16142549

**Published:** 2024-07-15

**Authors:** Evgenia Kotsifa, Nikolaos Machairas, Apostolos Angelis, Nikolaos I. Nikiteas, Dimitrios Dimitroulis, Georgios C. Sotiropoulos

**Affiliations:** 2nd Propaedeutic Department of Surgery, General Hospital of Athens “Laiko”, National and Kapodistrian University of Athens, Agiou Thoma 17, 11527 Athens, Greece

**Keywords:** hepatocellular carcinoma, inflammation-based scores, prognostic factors

## Abstract

**Simple Summary:**

This review explores the emerging area of the prognostic significance and therapeutic implications of inflammation-based scores in Hepatocellular carcinoma (HCC). Through comprehensive analysis and synthesis of existing literature, the paper highlights the prognostic value of these scores in predicting survival outcomes, recurrence, and treatment response in HCC patients. Furthermore, it discusses the underlying mechanisms linking inflammation with HCC progression and explores potential implications for personalized therapeutic strategies. Overall, this review underscores the importance of integrating inflammation-based scores into clinical practice for better risk stratification and management of HCC patients.

**Abstract:**

Hepatocellular carcinoma (HCC) is the most common primary liver cancer, posing a significant global health challenge with an increasing incidence. In recent years, multiple staging systems and scores have been proposed, emphasising the necessity for the development of precise prognostic tools. The well-documented etiological relationship between chronic inflammation and carcinogenesis has prompted researchers to explore novel prognostic markers associated with the inflammatory status of HCC patients. This review summarises the current data about inflammation-based scores in the context of HCC. We discuss established scores like the Glasgow Prognostic Score (GPS), modified GPS (mGPS) and the neutrophil-to-lymphocyte ratio (NLR) and others not as extensively studied, examining their utility in predicting survival outcomes and treatment response in HCC patients. Furthermore, we explore emerging scores, including the prognostic nutritional index (PNI) and other lymphocyte-based scores, assessing their potential in refining risk stratification and guiding therapeutic decisions in the era of precision medicine. As research progresses and these scores undergo further refinement and integration into the evolving landscape of HCC management, they carry significant potential for improving patient outcomes.

## 1. Introduction

Hepatocellular carcinoma (HCC) is the most common primary liver cancer, posing a significant global health challenge with an increasing incidence [1]. HCC is characterized by a multifactorial aetiology, often rooted in chronic liver diseases such as cirrhosis [2]. Approximately one-third of individuals with cirrhosis will inevitably develop HCC during their lifetime, exhibiting an annual incidence ranging from 1% to 8% [3]. As a disease of global significance, HCC’s prevalence varies across regions, reflecting disparities in risk factors, healthcare infrastructure, diagnostic tests, and access to preventive measures. Principal risk factors associated with HCC development include viral infections, specifically hepatitis B and C viruses (HBV, HCV), alcoholic liver disease (ALD), and non-alcoholic fatty liver disease (NAFLD) [4]. Viral hepatitis stands out as the predominant risk factor for HCC [5]. Despite this, organized vaccination programs against HBV and antiviral therapies for HBV/HCV have effectively curtailed HCC incidence in some regions. Conversely, the prevalence of HCC stemming from cirrhosis induced by NAFLD continues to rise, now constituting the primary cause of HCC in developed nations [6].

Treatment options for patients with HCC vary depending on multiple variables such as tumour burden, liver function and patient’s performance status [7]. Treatment modalities may include surgical resection, transplantation, locoregional treatments such as ablation, transarterial chemoembolization (TACE), and radioembolization (TARE), as well as systemic therapy or a combination of the above [8]. Finding the most appropriate treatment for patients with HCC can be a complex task, often requiring a multidisciplinary team to achieve the optimal outcome. Surgical treatment, including resection and transplantation, represents the ideal option for cirrhotic patients presenting with early-stage (BCLC stage 0 and A) HCC. Hepatectomy is typically considered for patients with small single lesions, preserved liver function, and good performance status [9]. Liver transplantation (LT) is optimal for patients with cirrhosis and portal hypertension [10]. The Milan criteria, established by Mazzaferro et al. in 1996, suggest that patients should only be considered for LT in the presence of a solitary tumour <5 cm, or up to three nodules with the largest measuring less than 3 cm, in the absence of vascular invasion or extrahepatic spread [11]. Although the Milan criteria have been subject to doubt and criticism, they continue to be implemented in clinical practice [12]. The AGMA score, a prognostic score for patients undergoing liver transplant for HCC, has also been proposed, excluding the tumour number/size criterion [13]. Locoregional treatments such as radio-frequency ablation (RFA), microwave ablation (MWA), and arterially directed therapies (TACE and TARE) are primarily available as options for patients who are not suitable candidates for surgery, but also as bridging therapies, as downstaging therapies, or in palliative settings. Arterially directed therapies are the first-line treatment for patients with intermediate-stage, characterized by multinodular HCC, relatively preserved liver function (Child–Pugh class A or B-7 points without ascites), absence of cancer-related symptoms and no evidence of vascular invasion or extrahepatic spread [14]. Finally, for patients presenting in an advanced stage with portal vein invasion and/or extrahepatic spread and poor performance status, systemic therapy with the use of oral multi-kinase inhibitors and immunotherapy with immune-checkpoint inhibitors can improve overall survival [15]. Patients in the terminal stage can be offered the best supportive care.

In recent years, multiple staging systems, biomarkers, and scores have been proposed, emphasising the necessity for the development of precise prognostic tools in patients undergoing liver resection for HCC [16,17,18]. These scores are created by a plethora of clinical, pathological, and biochemical markers and have merged as indispensable instruments in navigating the course of their disease [19]. Some of the most frequently used scoring systems include the tumour, node, and metastasis (TNM) system [20], the Barcelona Clinic Liver Cancer score (BCLC) [21], the Okuda system [22], the Japan Integrated Staging Score (JIS score) [23] and the Cancer of the Liver Italian Program (CLIP) [24]. These scores use multiple parameters related to tumour burden (tumour size and number), liver function (albumin and bilirubin levels) and the clinical condition of the patient (ascites and encephalopathy). More recently, the albumin–bilirubin (ALBI) grade has been developed, taking into consideration only aspects of the liver function [25]. However, the existence of numerous different scoring systems and the challenge of implementing a single, universal system manifest that none of the existing scores are perfect as well as the heterogeneity among HCC patients and the need for a personalized approach.

This review summarises the current data about inflammation-based scores in the context of HCC, shedding light on their utility as prognostic indicators and their potential to guide clinical decision-making for HCC patients.

## 2. The Inflammation-Based Scores for HCC

### 2.1. Rationale of Development of Inflammation-Based Scores

The well-documented etiological relationship between chronic inflammation and carcinogenesis has prompted researchers to explore novel prognostic markers associated with the inflammatory status of HCC patients [26]. Over the past years, inflammation-based scores have been developed as valuable tools in the field, providing clinicians with a novel understanding of the systemic inflammatory response and its implications for disease progression and patient outcomes [27]. These scores, often derived from routine blood-based markers, offer a non-invasive means of stratifying HCC patients based on their inflammatory profiles, thereby aiding in the refinement of prognostic assessments and guiding personalized treatment strategies. Table 1 describes all the available inflammation-based prognostic scores for HCC that we found through a PubMed search.

### 2.2. Available Inflammation-Based Scores

#### 2.2.1. GPS, mGPS and neo-GPS

The Glasgow Prognostic Score (GPS) was the first inflammation-based scoring system, and was initially developed as a prognostic tool for patients with inoperable non-small-cell lung cancer [28]. It is a cumulative score based on C-reactive protein (CRP) and albumin (Alb) (Table 2). Since its initial use as a prognostic tool, the GPS has also been implemented in other types of cancer, such as HCC, whilst multiple studies have validated its efficacy as a clinically important prognostic system [46]. Compared to the other inflammation-based scores, GPS is the most studied and most commonly used. Ishizuka et al., in two different studies, concluded that the GPS is an important predictor of postoperative mortality in HCC patients undergoing hepatectomy and that it can also be used to further classify patients with low CLIP scores into three independent groups, serving as a staging tool [47,48]. Recently, another study concluded that GPS can even be used in the preoperative setting to predict overall and disease-free survival of HCC patients undergoing liver resection [49]. Additionally, this scoring system has been validated in patients with unresectable HCC treated with systemic therapy. In a study by Tada et al., GPS was found to be independently associated with overall survival and progression-free survival in these patients [50]. Finally, in comparison to other inflammation-based scores, GPS has demonstrated its superiority by showing a higher C-index than other inflammation-based scores such as the modified GPS, neutrophil to lymphocyte ratio (NLR), platelet to lymphocyte ratio (PLR), prognostic index (PI) and prognostic nutritional index (PNI) (0.608 vs. ≤0.532) [26,51].

A few years after the initial development of GPS, the authors proposed a modification of the score: the modified GPS (mGPS) [29]. They observed that patients with a score of 1 were primarily due to an elevated CRP concentration. Nevertheless, patients with a GPS of 1 due to hypoalbuminemia, had a higher 3-year overall survival rate compared to patients with a GPS of one due to an elevated CRP level (*p* = 0.0094). Therefore, the GPS was modified such that patients with hypoalbuminemia were assigned a score of 0 in the absence of an elevated CRP level. Due to its resemblance to the original score, the mGPS has also been validated in multiple studies [26,52], with one of them concluding its superiority over other scores [53].

Recently, the neo-GPS has been developed, using ALBI grade instead of serum albumin as it is considered a more relevant nutrition marker for patients with chronic liver disease [30]. The reason for this is that the ALBI score has been shown to discern the borderline amino acid imbalance observed in cirrhotic patients [54]. The authors who developed the new score concluded that neo-GPS demonstrates a better predictive value for prognosis but also shows greater sensitivity for predicting the risk of postoperative complications as compared to GPS in patients undergoing hepatectomy for HCC [30]. Neo-GPS has also been validated as a prognostic tool in patients with advanced unresectable tumours receiving immunotherapy [55]. To our knowledge, only one study has addressed the comparison of neo-GPS to other inflammation-based scores and concluded that the c-indexes for the predictive prognostic value for overall survival and progression-free survival were higher for neo-GPS compared to other scores (0.571 vs. ≤0.555 and 0.555 vs. ≤0.546, respectively) [56]. Due to the score’s recent development, further studies are needed to establish its superiority.

#### 2.2.2. CAR

The two parameters used to calculate GPS (CRP and albumin) have undergone further investigation, leading to the development of another score. The CRP/Alb ratio (CAR) was originally developed and utilised to predict outcomes in patients in acute medical wards as well as in septic patients in intensive care units (ICUs) [57,58]. Kinoshita et al. evaluated 186 patients with HCC who underwent surgical resection, ablation, and transarterial therapies and received systematic treatment or best-supporting care, depending on their stage [31]. The researchers concluded that an elevated pre-treatment CAR (≥0.037) was associated with a higher Child–Pugh grade (*p* = 0.002), higher CLIP score (*p* < 0.0001), higher BCLC stage (*p* < 0.0001), larger tumour size (*p* < 0.0001), multiple nodules (*p* = 0.003), the presence of vascular invasion (*p* < 0.0001), and extrahepatic metastasis (*p* = 0.009). Additionally, CAR was independently associated with overall survival (hazard ratio 3.394; *p* < 0.0001) [31]. 

Another study investigated the association between postoperative high-sensitivity CRP (hsCRP)/albumin ratio and both overall survival and recurrence-free survival in 389 HCC patients treated with hepatectomy [59]. The study concluded that a postoperative hsCRP/albumin ratio increase of 1.0 was associated with a 1.171-fold increase in mortality (hazard ratio (HR): 1.171, 95% confidence interval (CI): 1.072–1.278, *p* < 0.001) and a 1.19-fold increase in recurrence (HR: 1.190, 95% CI: 1.108–1.278, *p* < 0.001) [59]. 

Finally, CAR has been also used to predict outcomes in patients with unresectable HCC treated with lenvatinib. Tada et al. concluded that a high CAR (≥ 0.108) was independently associated with overall survival (hazard ratio (HR), 1.915; 95% CI, 1.495–2.452) and progression-free survival (HR, 1.644; 95% CI, 1.324–2.043) [60].

#### 2.2.3. PI and PNI

The prognostic index (PI), calculated using a combination of CRP and white cell count, was initially explored as a prognostic marker in patients with lung cancer (Table 3). In those patients, an elevated PI was independently associated with disease progression in response to chemotherapy and a shorter overall survival [32]. 

Concerning the relation between PI and HCC, two studies investigating the prognostic value of multiple inflammation-based scores concluded that an elevated PI was associated with reduced overall survival and post-recurrence survival (*p* < 0.05) [51,61]. However, in comparison with other scores, it was found that these alternative scores were superior to PI. As a result, the clinical significance of PI remains low.

The prognostic nutritional index (PNI) is calculated by the sum of albumin and the total lymphocyte count (Table 3). In various studies, PNI has been shown to be a useful prognostic tool for multiple gastrointestinal malignancies [62,63]. More recently, in a large study by Proctor et al., the PNI was found to predict prognosis in malignancy regardless of the site of origin [64]. 

Regarding HCC, PNI has been established as an independent predictor of overall survival and recurrence-free survival in multiple studies and it has even proven to be superior to other scores [33,65,66]. In addition, PNI can serve as an independent prognostic factor for overall survival and progression-free survival in patients with advanced HCC treated with atezolizumab plus bevacizumab [67].

Finally, the above has been also validated by a meta-analysis that concluded that a lower level of preoperative PNI was a significant predictor of worse overall survival (HR = 1.82, 95% CI: 1.44–2.31) and disease-free survival (HR = 1.49, 95% CI: 1.06–2.07). In addition, a lower preoperative PNI was associated with an increased risk of postoperative recurrence (OR = 1.92, 95% CI: 1.33–2.76) [68]. 

#### 2.2.4. Lymphocyte Count-Based Scores

Lymphocyte count, a significant component of the immune system, has been used over the past years as a parameter of inflammatory status, which affects prognosis in cancer patients [69]. When combined with other parameters of the tumour immune microenvironment such as the neutrophil count (neutrophil to lymphocyte ratio—NLR), the platelet count (platelet to lymphocyte ratio—PLR) and the monocyte count (monocyte to lymphocyte ratio—MLR or Lymphocyte to Monocyte ratio—LMR), the derived scores serve as combined factors of inflammation and host immune reaction. They offer additional insights into a cancer patient’s prognosis and a more comprehensive assessment of the patient’s immune status, inflammatory response, and overall disease state.

Cancer patients often present a relative lymphocytopenia, which translates into a weak lymphocyte-mediated immune response to tumours, allowing cancer cells to proliferate and spread more easily. Additionally, since lymphocytes aid in the elimination of abnormal cells, a reduced lymphocyte count can result in impaired surveillance, allowing cancer cells to escape detection and evade the immune system [34,70]. On the contrary, cancer patients also display increased counts of neutrophils, leading to higher secretion of pro-angiogenic factors, providing an adequate environment for tumour growth and even metastasis via angiogenesis [71,72,73]. The above suggests the establishment of an imbalance between pro-inflammatory and anti-tumour immune responses, resulting in tumour progression and subsequently a poor prognosis.

NLR is the most commonly used lymphocyte count-based score and numerous studies have validated its significance as a prognostic factor, though the optimal cut-off value remains ill-determined. Yang et al. retrospectively reviewed 526 patients with HCC who underwent surgery. They showed that a preoperative NLR ≥ 2.81 was an independent predictor of poor disease-free survival (DFS, *p* < 0.001) and overall survival (OS, *p* = 0.044). They also noted that patients showing a postoperative decrease in NLR exhibited better survival rates [34]. In another study by Okamura et al., conducted retrospectively in 256 HCC patients, NLR (with a cut-off value of 2.81) was found to be an independent prognostic factor for overall and recurrence-free survival (hazard ratio [HR] 2.59, 95% confidence interval [CI] 1.56–4.31, *p* < 0.001, and HR 2.11, 95% CI 1.44–3.11, *p* < 0.001, respectively) [74]. Liao et al. concluded that a preoperative NLR value > 2.31 was an adverse predictor of DFS and OS in HCC after hepatectomy [73]. Gomez et al. reached the same conclusion but set the cut-off value higher (NLR ≥ 5) [75]. The above findings were confirmed by a recent metanalysis [76]. 

Thrombocytosis in cancer patients is a common finding due to several mechanisms that result in platelet activation and subsequent aggregation [77]. Platelets, typically known for their role in hemostasis and wound healing, also play a significant role in cancer progression. Platelets can release various inflammatory mediators and growth factors, including TGF-β and VEGF, which promote tumour cell proliferation, differentiation and angiogenesis. They also help tumour cells survive in the bloodstream by forming protective aggregates around them, known as tumour-platelet aggregates, facilitating metastasis [78]. Finally, platelets can interact with immune cells, such as T cells and natural killer (NK) cells, promoting an immunosuppressive environment that protects tumour cells from immunosurveillance [79].

Again, numerous studies have highlighted the importance of PLR as a significant prognostic factor in predicting outcomes in HCC patients [35,78,80,81].

Monocytes have recently also emerged as important regulators of tumour development [82]. Even though their relationship with cancer involves both pro-tumour and anti-tumour effects, it is thought that circulating monocytes are recruited to the tumour microenvironment and differentiate into tumour-associated macrophages (TAMs) which promote tumour growth by producing various cytokines and growth factors [83]. Monocytes also suppress T cell function, escaping from anti-tumour immune responses and promoting angiogenesis by releasing pro-angiogenic factors [84].

Even though MLR and LMR are not as extensively studied as the other lymphocyte-based scores, several studies have validated their importance as prognostic factors [36,85,86,87].

Finally, the lymphocyte-CRP ratio (LCR), the most recent lymphocyte-based score, combining aspects of both the immune system and inflammation, has gained ground in HCC prognosis research, though further research is needed to validate its utility and establish standardized cut-off values for clinical use [37,88,89,90,91].

#### 2.2.5. SII, SIRI, IINS, and ILIS

The systemic immune inflammation index (SII) is a prognostic score based on lymphocyte, neutrophil, and platelet counts in peripheral blood (SII = P × N/L, where P, N, and L are the peripheral platelets, neutrophil, and lymphocyte counts, respectively) [38]. The research team that developed the score retrospectively studied 133 HCC patients undergoing hepatectomy. The researchers concluded that the SII was an independent predictor for overall survival and relapse-free survival and that SII ≥ 330 was significantly associated with vascular invasion, large tumours, and early recurrence. Two other studies by Wang et al. and Li et al. confirmed the above results but set different cut-off values (461.5 and 0.728, respectively) [66,92]. Finally, a meta-analysis assessing the prognostic impact of pre-treatment SII concluded that an elevated SII was a poor prognostic factor for HCC patients [93].

The idea of combining three peripheral blood cells led to the development of another score named the Systemic Inflammation Response Index (SIRI) [94]. SIRI (calculated as follows: N × M/L, where N, M, and L are the peripheral neutrophil, monocyte, and lymphocyte counts, respectively) was originally created to predict the survival of patients with pancreatic cancer after chemotherapy [95]. Although certain studies have addressed the role of SIRI as a prognostic factor for HCC patients [96,97], SIRI is less extensively studied compared to other inflammation-based scores, and further studies are needed to determine its clinical significance.

The Inflammation-Immunity-Nutrition Score (IINS), originally developed as a prognostic score for patients with colorectal cancer [39], was constructed based on the sum of classification scores of preoperative high-sensitivity C-reactive protein (hsCRP), lymphocyte (LYM), and albumin (ALB). To our knowledge, only one study assessing the prognostic significance of IINS in HCC exists. According to that study, patients with a high preoperative IINS showed significantly worse OS and PFS compared to the low IINS group patients. Based on that result, the study concluded that the IINS could be a powerful clinical prognostic indicator in HCC patients undergoing radical surgery [98].

The Integrated Liver Inflammatory Score (ILIS) is an HCC-specific prognostic index built on 5 blood parameters (albumin, bilirubin, ALP, neutrophil count and AFP) calculated as follows: 0.057 × albumin (g/L) + 0.978 × log (Bilirubin, µmol/L) + 1.341 × log (ALP, IU/L) + 0.086 × neutrophil (10^9^/L) + 0.301 × log (AFP, µg/L). The researchers who developed the score categorized patients into three groups according to their score and concluded that ILIS was an independent prognostic factor for overall survival, which was validated in both Eastern and Western populations of HCC [40]. Another study group confirmed the above by studying 432 HCC patients undergoing radical hepatectomy. They concluded that ILIS was correlated with pathological features such as tumour size, degree of differentiation, Child–Pugh class classification, and BCLC staging, and that ILIS can serve as an independent risk factor for OS [99]. Nevertheless, the association between ILIS and HCC prognosis needs to be verified by further high-volume studies.

#### 2.2.6. ALRI, APRI, and ANRI

Aspartate aminotransferase (AST), also known as serum glutamic oxaloacetic transaminase (SGOT), is an enzyme found in various tissues of the body, with notable concentrations in the liver, heart, skeletal muscle and kidneys. Specifically, the liver-originated enzyme is released into the bloodstream primarily in response to liver damage or inflammation, making AST is significant marker of liver function assessment.

The rationale behind using the AST to lymphocyte ratio index (ALRI) is that it reflects both the inflammatory and immune status of the body. In the context of HCC, elevated AST levels may indicate liver damage, while a decreased lymphocyte count may signal compromised immune function. In a retrospective study by Jin et al., 371 HCC patients treated with hepatectomy were reviewed. The study concluded that elevated preoperative ALRI (>25.2) emerged as an independent prognostic factor associated with both poorer disease-free survival and overall survival when compared to patients with low preoperative ALRI (≤25.2) [41]. ALRI has also been investigated in a postoperative setting, in HCC patients undergoing radical liver resection. The study’s outcomes demonstrated that ALRI serves as an effective predictive marker for resected HCC patients, with the cut-off value determined at 22.6 [100]. A similar conclusion was drawn in another study involving HCC patients treated with palliative treatments. In this study population, the threshold value of ALRI was determined to be 86.3 [101].

AST has also been integrated into platelet counts to create the AST to platelet ratio index (APRI), which is a non-invasive marker primarily employed for assessing the degree of liver fibrosis and cirrhosis [102]. It is often used as part of the evaluation of liver fibrosis in individuals with chronic liver diseases, particularly hepatitis C [103]. However, APRI has also been demonstrated utility as a prognostic score for HCC. A retrospective study including 332 HCC patients treated with hepatectomy concluded that patients with preoperative APRI <0.62 exhibited significantly better disease-free survival and overall survival compared to patients with an elevated APRI (*p* = 0.009 and 0.002, respectively). In addition, elevated APRI values were associated with cirrhosis, hepatitis B virus (HBV) infection, surgical margin <1 cm, and non-capsulated tumours [43]. Another study assessing the ability of APRI to predict the risk of early mortality in HCC patients concluded that higher APRI values [odds ratio (OR) 1.33, 95% confidence interval (CI): 1.03–1.71, *p* = 0.03] were predictive of <30-day death and worse overall survival [hazard ratio (HR) 1.15, 95% CI: 1.03–1.30, *p* = 0.02] [104]. Finally, the authors suggested that APRI may be valuable to stratify high-risk patients when considering invasive procedures.

A third prognostic score involving AST is the AST to neutrophil ratio index (ANRI). To our knowledge, only one study has investigated the association between ANRI and prognosis in HCC. In this retrospective study, 303 patients who underwent curative resection for HCC were included. The study’s findings indicated that preoperative ANRI levels > 7.8 were correlated with a poor survival outcome. Specifically, the 1-, 3-, and 5-year disease-free survival rates of the ANRI ≤ 7.8 group were significantly higher than those of the ANRI > 7.8 group (59.8%, 46.7%, and 43.3% vs. 40.8%, 23.7%, and 20.2%, respectively, *p* < 0.001) while the 1-, 3-, and 5-year overall survival rates of the ANRI ≤ 7.8 group were also markedly higher than those of the ANRI > 7.8 group (81.5%, 62.0%, and 55.4% vs. 70.6%, 39.3%, and 31.6%, respectively, *p* < 0.001). The study also highlighted the significant prognostic value of preoperative ANRI in patients with TNM stage I. Consequently, the researchers concluded that preoperative ANRI could be utilized as a predictive marker for recurrence in early HCC [42].

#### 2.2.7. FAR and PAL

Fibrinogen is a soluble plasma glycoprotein synthesized in the liver, and is one of the key components involved in blood clotting. Its primary role is to help in the formation of blood clots, which is crucial for wound healing and preventing excessive bleeding when tissues are damaged [105]. Fibrinogen is also considered an acute-phase protein; therefore, its levels in the blood can increase dramatically in response to inflammation or tissue injury. Fibrinogen and its degradation products can interact with various cells and molecules involved in inflammation, including leukocytes, endothelial cells, and cytokines. These interactions can modulate the inflammatory response, influencing processes such as leukocyte recruitment, cytokine production, and tissue remodelling [106]. Considering its function as an acute-phase molecule, fibrinogen has been combined with albumin to form a ratio (fibrinogen/albumin ratio—FAR). The researchers who created it calculated retrospectively the preoperative fibrinogen/albumin ratio of 150 HCC patients who underwent curative resection. They concluded that a FAR > 0.062 was significantly associated with microvascular invasion, an advanced BCLC stage, and ALBI grade. Additionally, multivariate analyses revealed that FAR was an independent predictor for overall survival (*p* = 0.003) and time to recurrence (*p* = 0.035) [44].

A second score involving albumin levels is the platelet–albumin score (PAL). Shindoh et al. proposed this new score based on a cohort of 889 HCC patients who underwent surgical resection. The results of the study revealed that PAL could predict not only the overall survival but also the postoperative outcomes of those patients. Hence, it can be used as a grading system for the stratification of survival outcomes and surgical risks of patients undergoing HCC resection [45]. Recently, these findings were validated in the Western population. Specifically, a higher PAL grade is significantly associated with higher postoperative morbidity (*p* = 0.039), post-hepatectomy liver failure (*p* = 0.001), perioperative mortality (*p* = 0.036), and overall survival (*p* = 0.018) [107].

### 2.3. Combinations

After the development and establishment of some of the aforementioned scores, new combinations of inflammation-based scores have emerged. These combinations may provide a broader understanding of the interplay between systemic inflammation, tumour biology and patient outcomes, thereby facilitating refined risk stratification and tailored therapeutic strategies. By using the synergistic information obtained from multiple scoring systems, clinicians can optimize patient management approaches, potentially improving survival outcomes and treatment efficacy in HCC. However, conducting an in-depth analysis of all existing combined scores is beyond the scope of this review. Interested readers can find some examples in Table 4.

## 3. Discussion

Our review sought to analyse the existing literature on the prognostic significance and therapeutic implications of inflammation-based scores in HCC. The reviewed studies have demonstrated the association of these scores with overall and disease-free survival as well as response to therapy. Furthermore, inflammation-based scores have been proven as independent prognostic markers, aiding in risk stratification and personalized treatment approaches.

As the field of immunotherapy gains prominence in HCC treatment, the integration of inflammation-based scores with immunotherapeutic strategies presents an exciting avenue for further exploration. Given the notable variance in the efficacy of immunotherapy among individuals, there is a growing imperative for the emergence of reliable prognostic indicators. A recent study, comparing the prognostic value of various inflammation-based scores in HCC patients after anti-PD-1 therapy, concluded that all the studied inflammation-based scores showed strong discriminatory ability in overall survival [114]. This study, despite its limitations, represents the first to propose the use of inflammation-based scores as potential decision-guiding tools for HCC patients receiving immunotherapy.

While immunotherapy is presently administered within the palliative setting, current studies are examining its potential application as a neoadjuvant intervention in unresectable hepatocellular carcinoma, serving as a downstaging modality [115]. Moreover, in resectable HCC cases, it may be employed to minimize relapse occurrences and enhance overall survival rates when utilized as both adjuvant and neo-adjuvant treatment [116,117]. The IMbrave050 trial most recently showed a significant benefit in terms of DFS for patients at high risk of HCC recurrence following curative-intent resection or ablation, who received adjuvant atezolizumab plus bevacizumab compared to active surveillance [118]. Thus, patients undergoing surgery for resectable HCC with high inflammatory scores and a theoretically higher risk for recurrence could be promptly recognized and administered adjuvant immunotherapy. 

Despite their promise, challenges remain in the widespread adoption of inflammation-based scores in clinical practice. Standardization of scoring systems, integration of underlying liver disease aetiology, and external validation across diverse patient groups are crucial for increasing their reliability and reproducibility. While inflammation-based scores offer valuable prognostic information in HCC, they should be interpreted alongside other clinical factors and staging systems. Integrating inflammation-based scores with established staging systems, such as the BCLC and CLIP, can enhance prognostic accuracy and help optimize treatment strategies for HCC patients [26,119,120]. 

Another limitation of these scores is that they are all derived through retrospective analysis, relying on data collected from past patient cohorts, which may introduce biases and limitations in the interpretation of results. Due to the lack of prospective studies and randomised control trials, it is not possible to assess the credibility of each score and compare them effectively. Furthermore, a significant proportion of these studies fail to clearly specify whether the studied population comprises cirrhotic or non-cirrhotic patients, rendering it challenging to generalize findings across different patient cohorts. Moreover, the aetiology of cirrhosis, a crucial determinant of inflammatory status and disease progression, is often overlooked in these analyses. This oversight can potentially confound results, as the inflammatory landscape varies considerably among patients with different underlying liver diseases. Consequently, while inflammation-based scores offer valuable insights into the prognostic landscape of HCC, their application must be approached with caution considering these inherent limitations. 

Regarding the comparison of the scores, it is important to note that the majority of these scores have been developed and validated in single-centre, retrospective studies using different patient populations. There is a significant variation in the populations studied, methodologies used, and clinical settings, making direct comparisons challenging. Moreover, there is currently no study that systematically compares all the available scores to determine which is the most effective. This variability in the data prevents a definitive conclusion on the superiority of one score over another.

## 4. Conclusions

In conclusion, inflammation-based scores in HCC provide valuable insights into the well-documented relationship between inflammation and cancer progression. Leveraging the inflammation-based scores may enable clinicians to identify patients with increased inflammatory states, potentially indicating high-risk individuals who may derive significant benefits from perioperative immunotherapy interventions. Although further studies are needed to validate these results, understanding the dynamic interplay between inflammation and immune response will likely refine prognostic models, guiding a more personalized approach. 

Their prognostic significance highlights their potential as valuable clinical tools for risk stratification and treatment planning. As research progresses and these scores undergo further refinement and integration into the evolving landscape of HCC management, they carry significant potential for improving patient outcomes; nonetheless, further prospective studies are warranted to ensure their incorporation in official guidelines for the management of patients with HCC eligible for resection.

## Figures and Tables

**Table 1:** Inflammation-based prognostic scores.

	AFP	Bil	Alb	ALP	AST	CRP	Fbg	WBC	Neutrophils	PLTs	Monocytes	Lymphocytes
GPS [28]			√			√						
mGPS [29]			√			√						
Neo-GPS [30]		√	√			√						
CAR [31]			√			√						
PI [32]						√		√				
PNI [33]			√									√
NLR [34]									√			√
PLR [35]										√		√
MLR [36]											√	√
LCR [37]						√						√
SII [38]									√	√		√
SIRI [38]									√		√	√
IINS [39]			√			√						√
ILIS [40]	√	√	√	√					√			
ALRI [41]					√							√
ANRI [42]					√				√			
APRI [43]					√					√		
FAR [44]			√				√					
PAL [45]			√							√		

AFP: alpha-fetoprotein, Bil: Bilirubin, Alb: Albumin, ALP: alkaline phosphatase, AST: aspartate transaminase, CRP: c-reactive protein, Fbg: fibrinogen, WBC: white blood count, PLTs: platelets, GPS: Glasgow prognostic score, mGPS: modified Glasgow prognostic score, CAR: c-reactive protein to albumin ratio, PI: prognostic index, PNI: prognostic nutritional index, NLR: neutrophil to lymphocyte ratio, PLR: platelet to lymphocyte ratio, MLR: monocyte to lymphocyte ratio, LCR: lymphocyte to c-reactive protein ratio, SII: systemic immune inflammation index, SIRI: Systemic Inflammation Response Index, IINS: Inflammation-Immunity-Nutrition Score, ILIS: Integrated Liver Inflammatory Score, ALRI: aspartate aminotransferase to lymphocyte ratio index, ANRI: aspartate aminotransferase to neutrophil ratio, APRI: aspartate aminotransferase to platelet ratio index, FAR: fibrinogen to albumin ratio, PAL: platelet–albumin score.

**Table 2:** Calculation of GPS and mGPS. CRP: C-reactive protein, Alb: albumin.

GPS	Score
CRP ≤ 10 mg/L and Alb ≥ 35 g/L	0
CRP ≤ 10 mg/L and Alb < 35 g/L	1
CRP > 10 mg/L and Alb ≥ 35 g/L	1
CRP > 10 mg/L and Alb < 35 g/L	2
mGPS	
CRP ≤ 10 mg/L and any Alb	0
CRP > 10 mg/L and Alb ≥ 35 g/L	1
CRP > 10 mg/L and Alb < 35 g/L	2

**Table 3:** Calculation of PI and PNI.

Prognostic Index (PI)	Score
CRP ≤ 10 mg/L and WBC ≤ 11 × 10^9^/L	0
CRP ≤ 10 mg/L and WBC > 11 × 10^9^/L	1
CRP > 10 mg/L and WBC ≤ 11 × 10^9^/L	1
CRP > 10 mg/L and WBC > 11 × 10^9^/L	2
Prognostic Nutritional Index (PNI)	
Alb (g/L) + 5 × total lymphocyte count (×10^9^/L) ≥ 45	0
Alb (g/L) + 5 × total lymphocyte count (×10^9^/L) < 45	1

CRP: C-reactive protein, Alb: albumin, WBC: white blood count.

**Table 4:** Combinations of inflammation-based scores.

	Alb	GGT	ALT	AST	Fbg	Hgb	WBC	Neutrophils	PLTs	Lymphocytes
F-NLR [108]					√			√		√
COP-NLR [109]								√	√	√
NLRAR [110]	√							√		√
WHR [110]						√	√			
NWS [110]	√					√	√	√		√
GPR/Fbg [111]		√			√				√	
PNI-APRI [112]	√			√					√	√
PNI-GGT/ALT [113]	√	√	√							√

Alb: Albumin, GGT: gamma-glutamyl transferase, ALT: alanine transaminase, AST: aspartate transaminase, Fbg: fibrinogen, Hgb: haemoglobin, WBC: white blood count, PLTs: platelets, F-NLR: fibrinogen and neutrophil to lymphocyte ratio, COP-NLR: platelet count and neutrophil to lymphocyte ratio, NLRAR: neutrophil to lymphocyte ratio to albumin ratio, WHR: white blood cell to haemoglobin ratio, NWS: NLRAR-WHR scoring system, GPR/Fbg: gamma-glutamyl transpeptidase to platelet ratio/Fibrinogen, PNI-APRI: prognostic nutritional index and aspartate aminotransferase to platelet ratio index, PNI-GGT/ALT: prognostic nutritional index-gamma-glutamyl transferase/alanine transaminase.
